# Novel Positively Charged Nanoparticle Labeling for *In Vivo* Imaging of Adipose Tissue-Derived Stem Cells

**DOI:** 10.1371/journal.pone.0110142

**Published:** 2014-11-03

**Authors:** Hiroshi Yukawa, Shingo Nakagawa, Yasuma Yoshizumi, Masaki Watanabe, Hiroaki Saito, Yoshitaka Miyamoto, Hirofumi Noguchi, Koichi Oishi, Kenji Ono, Makoto Sawada, Ichiro Kato, Daisuke Onoshima, Momoko Obayashi, Yumi Hayashi, Noritada Kaji, Tetsuya Ishikawa, Shuji Hayashi, Yoshinobu Baba

**Affiliations:** 1 Research Center for Innovative Nanobiodevices, Nagoya University, Furo-cho, Chikusa-ku, Nagoya 464-8603, Japan; 2 Department of Medical Technology, Nagoya University, Graduate School of Medicine, Daikominami, Higashi-ku, Nagoya 461-8673, Japan; 3 Department of Applied Chemistry, Graduate School of Engineering, Nagoya University, Furo-cho, Chikusa-ku, Nagoya 464-8603, Japan; 4 Nagoya Research Laboratory, MEITO Sangyo Co., Ltd., Kiyosu 452-0067, Japan; 5 Department of Advanced Medicine in Biotechnology and Robotics, Graduate School of Medicine, Nagoya University, Higashi-ku, Nagoya 461-0047, Japan; 6 Department of Regenerative Medicine, Graduate School of Medicine, University of the Ryukyus, 207 Uehara, Nishihara, Okinawa 903-0215, Japan; 7 Research Institute of Environmental Medicine, Stress Adaption and Protection, Nagoya University, Furo-cho, Chikusa-ku, Nagoya, 464-8601, Japan; 8 Institute of Innovative for Future Society, Nagoya University, Furo-cho, Chikusa-ku, Nagoya 464-8603, Japan; 9 Health Research Institute, National Institute of Advanced Industrial Science and Technology (AIST), Hayashi-cho 2217-14, Takamatsu 761-0395, Japan; Georgia Regents University, United States of America

## Abstract

Stem cell transplantation has been expected to have various applications for regenerative medicine. However, in order to detect and trace the transplanted stem cells in the body, non-invasive and widely clinically available cell imaging technologies are required. In this paper, we focused on magnetic resonance (MR) imaging technology, and investigated whether the trimethylamino dextran-coated magnetic iron oxide nanoparticle -03 (TMADM-03), which was newly developed by our group, could be used for labeling adipose tissue-derived stem cells (ASCs) as a contrast agent. No cytotoxicity was observed in ASCs transduced with less than 100 µg-Fe/mL of TMADM-03 after a one hour transduction time. The transduction efficiency of TMADM-03 into ASCs was about four-fold more efficient than that of the alkali-treated dextran-coated magnetic iron oxide nanoparticle (ATDM), which is a major component of commercially available contrast agents such as ferucarbotran (Resovist), and the level of labeling was maintained for at least two weeks. In addition, the differentiation ability of ASCs labeled with TMADM-03 and their ability to produce cytokines such as hepatocyte growth factor (HGF), vascular endothelial growth factor (VEGF) and prostaglandin E2 (PGE2), were confirmed to be maintained. The ASCs labeled with TMADM-03 were transplanted into the left kidney capsule of a mouse. The labeled ASCs could be imaged with good contrast using a 1T MR imaging system. These data suggest that TMADM-03 can therefore be utilized as a contrast agent for the MR imaging of stem cells.

## Introduction

Cell transplantation, which is a simple, rapid and minimally-invasive method relative to whole organ transplantation, has been demonstrated to be effective for treating various diseases such as diabetes, central nervous system (CNS) disorders and cancers including hematological diseases [1]. In particular, stem cell transplantation has been expected to have applications for regenerative medicine. Tsuji et al. showed that the transplantation of induced pluripotent stem (iPS) cells -derived neurospheres was effective for treating spinal cord injury [2]. Liu et al. showed that the transplantation of a combination of mesenchymal stromal cells and haploidentical hematopoietic stem cells facilitated platelet recovery without increasing the recurrence of leukemia [3]. However, the clinical application of stem cell transplantation for many internal organs has been restricted due to the lack of sufficient technology to trace such transplanted stem cells to confirm their correct implantation and to evaluate their growth and migration *in vivo* [4].

In order to reveal the location and accumulation of transplanted stem cells in various tissues and organs deep in the body, a non-invasive and widely clinically available cell imaging technology is needed [5], [6]. We herein focus on magnetic resonance (MR) imaging as a method for tracing the transplanted stem cells, because it is a non-invasive, irradiation-free and clinically used method offering good tissue contrast [7]. The MR imaging of stem cells is currently an emerging strategy for tracing transplanted stem cells. To increase the contrast of issues in typical imaging studies, MR contrast agents such as gadolinium (Gd) and superparamagnetic iron oxide (SPIO) nanoparticles are generally used [8], [9]. These agents cause hydrogen relaxivity changes and induce contrast modifications [8]. In particular, SPIO nanoparticles are known to generate a strong transverse relaxation time T2-negative contrast in MR images and to decrease the signal intensity [10]. In addition, T2-weighted agents including SPIO nanoparticles are preferentially used for cellular MR imaging since they are more biocompatible and more highly magnetic than T1-weighted agents, resulting in higher contrast modification on MR imaging with a lower concentration than T1-weighted agents [8].

Various SPIO nanoparticles have been developed as contrast agents, including ferucarbotran (Resovist), ferumoxide (Feridex, Endorem) and ferumoxtran-10 (Combidex, Sinerem) [11], [12]. Ferucarbotran, an anionic SPIO nanoparticle with a carboxydextran coating has been successfully applied in the clinical setting as a liver contrast agent [13]. It was recently reported that ferucarbotran could more efficiency magnetically label stem cells than ferumoxide and ferumoxtran without including cytotoxicity [4], [14]. In this study, we also demonstrate that stem cells can be labeled with ATDM which is a major component of ferucarbotran.

A more common method of labeling cells utilizes cationic transfection reagents to induce the formation of complexes with negatively charged SPIO nanoparticles, because positive charges have been generally considered to be effective for accelerating the intracellular incorporation of such particles [15]-[18]. Several groups have shown that protamine, which is a low molecular weight polycationic peptide approved by the U.S. FDA as an antidote for heparin anticoagulation, enhanced the uptake of ATDM into stem cells [19]. However, they could not form stable complexes with SPIO nanoparticles, and therefore, it is difficult to clarify the influence of these agents on stem cells [20].

In order to overcome these problems, we have developed five novel contrast agents to use for MR imaging; trimethylamino dextran-coated magnetic iron oxide nanoparticles with different positive charges [21], [22]. TMADM-03 has proven to be stably dispersed in the culture medium including fetal bovine serum, and is efficient for labeling mature cells without exerting cytotoxic effect. In fact, Min6 cells, which are a β-cell line, could be efficiently labeled with TMADM-03 without signs of cytotoxicity [22], [23]. However, the applicability of TMADM-03 for stem cells remains to be elucidated.

In our research group, adipose tissue-derived stem cells (ASCs) have been the major focus as the stem cell source for regenerative medicine, including stem cell transplantation [24]. ASCs can be easily obtained in abundance by minimally invasive harvest procedures, such as lipoaspiration under local anesthesia, and have the ability to differentiate into not only mesenchymal cells, but also epithelial and endothelial cells [25], [26]. Moreover, ASCs have already been used for some clinical treatments [27]. ASCs thus are expected to provide a useful and effective source of the stem cells for regenerative medicine, including stem cell transplantation.

In this study, we investigated whether TMADM-03 could efficiently label ASCs without adverse effects, and determined whether the labeled ASCs could be observed *in vitro* and *in vivo* using MR imaging.

## Materials and Methods

### Materials

ATDM, which is a major component of ferucarbotran (Resovist), and TMADM-03 were provided by Meito Sangyo Co., Ltd. (Nagoya, Japan). The Cell Counting Kit-8 (CCK-8) was purchased from Dojindo Laboratories (Kumamoto, Japan). Iron standard solution (Fe 1000) and LabAssay-triglyceride were purchased from Wako Pure Chemical Industries, Ltd. (Osaka, Japan). Microhomogenizers for 1.5 mL microtubes ((3810)226AG) were purchased from Eppendorf Japan (Tokyo, Japan). Inductively coupled plasma - atomic emission spectrometry (ICP-AES) was employed to measure the iron concentrations. The Adipo-Inducer Reagent and Osteoblast-Inducer Reagent were purchased from Takara Bio. Inc. (Shiga, Japan). The Quantikine Mouse HGF Immunoassay and Quantikine Mouse VEGF Immunoassay were purchased from R&D systems (Minneapolis, USA). The mouse PGE2 ELISA kit was purchased from Cusabio Biotech Co., Ltd. (Wuhan, China). MACS LS column was purchased from Miltenyi Biotech (Tokyo, Japan).

### Animals

C57BL/6 mice were purchased from SLC Japan. The mice were housed in a controlled environment (12 h light/dark cycles at 21°C) with free access to water and an alfalfa-free diet before sacrifice. All conditions and handing of animals in this study were conducted under protocols (024–002 and 025–018) approved by the Nagoya University Committee on Animal Use and Care.

### Isolation and culture of ASCs

The isolation and culture of ASCs were reported previously [26]. Briefly, ASCs were collected from seven to fourteen-month-old female C57BL/6 mice. The adipose tissues in the inguinal groove were isolated and cut finely, then digested with type II collagenase (Collagenase Type II, Koken Co., Ltd., Tokyo, Japan) at 37°C in a shaking water bath for 90 min. Adipose tissue cells were when suspended in culture medium (Dulbecco's modified Eagle's medium (DMEM)/F12 containing 20% fetal bovine serum (FBS: Trace Scientific Ltd., Melbourne, Australia) and 100 U/mL penicillin/streptomycin). The cells were centrifuged at 1,200 rpm for five minutes at room temperature to obtain a pellet containing the ASCs. The cells were washed three times by suspension and centrifugation in the culture medium. The primary cells were then cultured for four to five days until they reached confluence and were defined as passage “0”. The cells used in all of the experiments were between passages two and five.

### Cytotoxicity of ATDM and TMADM-03 to ASCs

ASCs (1×10^4^) were seeded in a 96-well plate (BD Biosciences) with 100 µL of culture medium for four hours at 37°C, which was then replaced with 100 µL of transduction medium (DMEM/F12 containing 2% FBS and 100 U/mL penicillin/streptomycin). ATDM (5 mg-Fe/mL) and TMADM-03 (5 mg-Fe/mL) were prepared at various concentrations (0, 5, 10, 50 and 100 µg-Fe/mL) with transduction medium, and were added into each well. After a one or 24 h incubation, the cells were counted using the CCK-8. The CCK-8 reagent (10 µL) was added to each well and the reaction was allowed to proceed for up to four hours. The absorbance of each sample at 450 nm was measured against a background control using a microplate reader.

### Proliferation of ASCs labeled with TMADM-03

ASCs (2×10^3^) were seeded in each well of a 96-well plate with 100 µL of culture medium and were incubated with various concentrations of TMADM-03. After one hour, the medium was changed to new incubation medium after the cells were washed with PBS three times to eliminate the remaining TMADM-03 in the culture medium. The cells were incubated for two or seven days, and then viable cells were counted using the CCK-8 in the same way as described above.

### Electron microscopy analysis

Electron microscopy was used to visualize the presence of TMADM-03 inside the ASCs. ASCs labeled with TMADM-03 were fixed with 2% paraformaldehyde and 2% glutaraldehyde in 0.1 M phosphate buffer (pH 7.4) at 4°C for 24 h, followed by incubation in 2% osmium tetroxide at 4°C for 90 min. The cells were dehydrated in increasing concentrations of ethanol, immersed in propylenoxide and then embedded in Quetol 812 (Nissin EM, Tokyo). Ultrathin sections (70 nm) were stained using Reynold's lead citrate and examined using a JEM-1200EX transmission electron microscope (TEM) (JOEL, Ltd., Tokyo) at an accelerating voltage of 80 kV. These studies were done in cooperation with the Tokai Electron Microscopy Analysis Co., Ltd. (Aichi, Japan).

### Quantitative determination of Fe in ASCs labeled with TMADM-03

ASCs (1×10^6^) were incubated with ATDM or TMADM-03 at various concentrations (10, 30 and 50 µg-Fe/mL) in transduction medium for one hour. The amount of Fe was measured by phenanthroline spectrophotometric method and ICP-AES method. Briefly, in the ICP-AES method, the ASCs labeled with ATDM or TMADM-03 were washed with PBS three times and were collected by trypsinization. Concentrated nitric acid solution (2 mL) was added to the collected cells, and thermolysis of the solution was conducted at 200°C for four to five hours. After the volatilization of the solution, distilled water was added to the pellets derived from the labeled cells until they weigh 5 g. Next, the Fe concentration of the pellets was measured using the ICP-AES at the analytical wavelength of 259.74 nm. Iron standard solution (Fe 1000) (Wako) was serially diluted, and then used as a standard solution of Fe for comparison purposes.

### The labeling efficiency of TMADM-03 for ASCs

The labeled ASCs with TMADM-03 were separated from unlabeled ASCs using MACS LS column in MACS technology according to the manufacture's procedure [28], [29]. In brief, ASCs (3×10^5^) were labeled with TMADM-03 (0, 10 and 30 µg-Fe/mL) in a one hour incubation, then the ASCs were washed with PBS three times and were collected by the centrifugation at 1200 rpm for 3 min. The ASCs were suspended with transduction medium (2 mL) and the cell suspension was filled into the prerinsed MACS LS column in the magnetic field of the MACS magnet. The ASCs labeled with TMADM-03 were bound to the column, whereas non-labeled ASCs passed through the column. The column was removed and the ASCs labeled with TMADM-03 were released from magnetic field. The column was washed three times with transduction medium (3 mL). The collected cells were counted, and the collected rate was calculated as the labeling efficiency of TMADM-03 for ASCs.

### Analysis of the mechanism of TMADM-03 uptake

ASCs (5×10^5^) were seeded in each well of a 6-well plate with 2 mL of culture medium and incubated for 24 h at 37°C. The cells were then treated with endocytosis inhibitors, 10 mM sodium azide and 2-deoxy-D-glucose, 5 mM amiloride, 5 µg filipin III, or 12.5 µg chlorpromazine (CPZ) at 37°C for one hour (15 min for amiloride), and then were treated with TMADM-03 (30 µg-Fe/mL) and incubated for one hour at 37°C. In addition, the treatment of incubation at 4°C for one hour was conducted to inhibit endocytosis. Then, the cells were collected and the Fe (II) concentration was measured as described above.

### Adipogenic differentiation

The Adipo-Inducer Reagent was used for the adipogenic differentiation of ASCs. Their differentiation was conducted in accordance with the accompanying product manual. Briefly, the differentiation solution was prepared by adding insulin solution (1 mL), dexamethasone solution (0.5 mL) and 3-isobuthyl-1-methylxanthine solution (0.1 mL) into the culture medium (100 mL). The incubation solution was prepared by adding insulin solution (1 mL) into the culture medium (100 mL). ASCs with or without the TMADM-03 label were incubated with the differentiation medium for two days. Thereafter, the medium was exchanged for the incubation medium, then cells were incubated for another five to ten days.

The adipogenic differentiation was confirmed by Oil Red O staining as an indicator of intracellular lipid accumulation. Briefly, the cells were fixed in a 10% solution of formaldehyde in PBS for at least 10 min at room temperature, and then were washed with 60% isopropanol. Next, the cells were stained with 2% (w/v) Oil Red O reagent for 10 min at room temperature, followed by repeated washing with distilled water and destaining in 100% isopropanol for one minute.

### Osteogenic differentiation

The Osteoblast-Inducer Reagent was used for the osteogenic differentiation of ASCs as specified by the manufacture's product manual. Briefly, the differentiation solution was prepared by adding ascorbic acid (1 mL), hydrocortisone (0.2 mL) and β-glycerophosphate (1 mL) into the culture medium (100 mL). ASCs with or without the TMADM-03 label were incubated with the differentiation medium for 14 to 21 days. The medium was changed to fresh differentiation medium every seven days.

The osteogenic differentiation was confirmed by staining for alkaline phosphatase activity. The cells were then washed twice with PBS and fixed in 10% formalin for 15 min at room temperature. They were then washed and incubated with deionized water for 15 min, and were subsequently stained with a solution containing naphthol AS MX-PO_4_ (Sigma, N-5000), *N, N*-dimethylformamide (Wako Pure Chemical Industries Ltd.), Red Violet LB salt (Sigma, F-1625) and Tris-HCl buffer (pH 8.3) for 45 min.

### Triglyceride measurement

ASCs (2×10^5^) were seeded in each well of a 12-well plate with 2 mL of culture medium and were transduced with TMADM-03 (30 µg-Fe/mL). After the process of adipogenic differentiation, the cells were treated with trypsin and collected into microtubes. PBS (100 µL) was added into the tubes, and then the cells were shredded with microhomogenizers. The amount of triglyceride in the microtubes was measured using the LabAssay Triglyceride Kit according to the manufacture's protocol. Briefly, the color-producing reagent was diluted with buffer solution and the coloring reagent was prepared. The coloring reagent was then added into the samples and standard solutions, and then incubated for five minutes at 37°C. The absorbance at 600 nm was measured by a BioPhotometer (Eppendorf, Tokyo, Japan) and the amount of triglyceride was calculated.

### Quantitative estimation of alkaline phosphatase expression

ASCs (1×10^5^) were seeded in each well of a 24-well plate with 1 mL of culture medium, and were transduced with TMADM-03 (30 µg-Fe/mL). After the process of osteogenic differentiation, the alkaline phosphatase expression was evaluated by measuring the alkaline phosphatase staining area.

### Enzyme-linked immunosorbent assays

ASCs (1×10^5^) were seeded into each well of a 24-well plate, and were transduced with TMADM-03 (30 µg-Fe/mL) for one hour. Then, the cells were washed with culture medium, and incubated in fresh culture medium for 24 or 72 h at 37°C. The culture supernatants were collected, and then the levels of mouse HGF, VEGF and PGE2 produced by the ASCs labeled with TMADM-03 and non-labeled ASCs (Normal) in the medium were measured using specific ELISA kits according to the manufacturer's protocols.

### ASCs transplantation

ASCs were labeled with TMADM-03 (30 µg-Fe/mL) for 1 h at 37°C. At the end of the uptake experiments, the ASCs were washed three times in the transduction medium, and were collected in microtubes. The ASCs (1×10^6^) were transplanted into the renal subcapsular space of the right kidney of a mouse. Moreover, the ASCs (2×10^6^) labeled with TMADM-03 (30 µg-Fe/mL) as the same condition were subcutaneously transplanted on the back of the mouse for *in vivo* imaging.

### MR imaging

The mice were lightly anesthetized using isoflurane (3% induction and 1.5% maintenance) prior to imaging. MR imaging data were collected on a 1T MRI (MRTechnology, Tsukuba, Japan) according to the manufacture's procedure. In brief, the imaging parameters were as follows: T2 sequences with a TR/TE of 3000/69 ms, field of view (FOV): 30 and two averages were taken for a total acquisition time of about 14 min. T1 sequences were composed of a TR/TE of 500/9 ms, FOV: 30 and four averages for a total acquisition time of about five minutes. All T1 and T2 -weighted image data sets were visually evaluated to identify the location of the transplanted cells within each animal.

### Prussian blue (PB) staining

The existence of iron particle (TMADM-03) within tissues was confirmed by the PB staining which is a traditional method for detecting the iron (ferric form) according to the manufacture's procedure [30], [31]. In brief, the hydrochloric acid and potassium ferrocyanide were mixed and prepared immediately before use. The slides were immersed in this solution for 20 min, and then washed in distilled water three times. Next, the slides were treated with counterstain solution with for 5 minutes, and the slides were rinsed twice in distilled water. Then, the slides were dehydrated through 95% to 100% alcohol, and cleared in xylene two times for 3 minutes each. The slides were covered with resinous mounting medium.

### Statistical analysis

Numerical values are presented as the means ± SD. Each experiment was repeated three times. The statistical significance was evaluated using unpaired Student's *t*-tests for comparisons between two groups; p-values <0.05 were considered to be statistically significant. All statistical analyses were performed using the SPSS software package.

## Results

### Cytotoxicity of ATDM and TMADM-03 to ASCs

**Figure 1:**
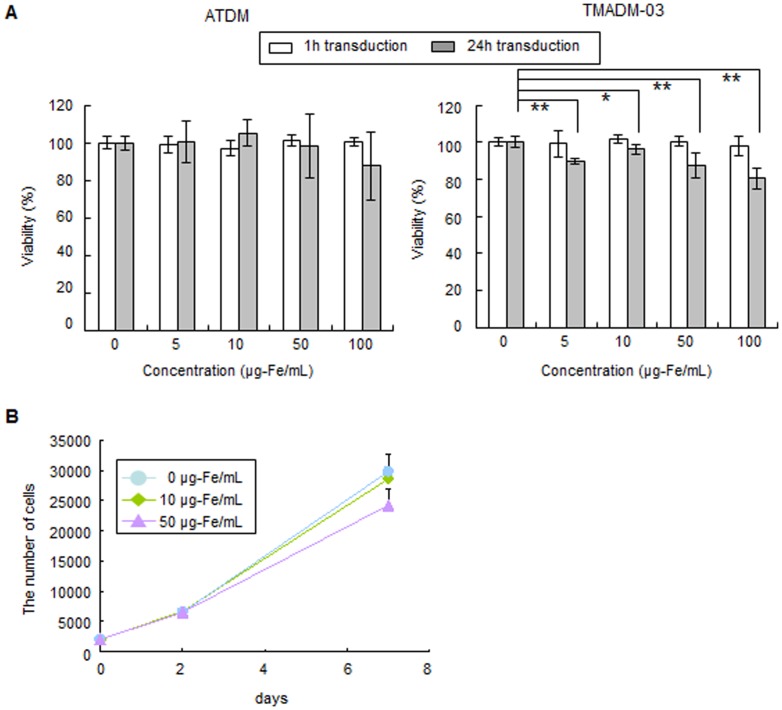
The viability and proliferation rate of ASCs labeled with ATDM or TMADM-03. A: The viability of ASCs labeled with ATDM or TMADM-03 (0, 5, 10, 50, 100 µg-Fe/cell) after a 1 h (white bars) or 24 h (gray bars) transduction at 37°C. There were significant differences in the viability of ASCs labeled with TMADM-03 after the 24 h transduction. B: The proliferation rate of ASCs labeled with TMADM-03 (0, 10, 50 µg-Fe/mL) at 0, 2 and 7 days after 1 h transduction. No significant differences were observed at any of the concentrations of TMADM-03. These data are shown as the means ± standard deviation of triplicate values. *P<0.05. **P<0.01.

ATDM and TMADM-03 were transduced into ASCs at various concentrations in transduction medium for one or 24 h incubations. Cytotoxicity was observed in the ASCs transduced for 24 h at all concentrations of TMADM-03, however, the degree of cytotoxicity was slight, and more than about 80% of the cells were still alive after the treatment. In addition, no cytotoxicity was observed after a one hour incubation at all concentrations of TMADM-03. On the other hand, no cytotoxicity of ATDM was observed under all of these experimental conditions (Fig. 1A).

Next, the influence of the compounds on the proliferation rate was examined for the non-cytotoxic conditions with TMADM-03. The cells were confirmed to exhibit a logarithmic growth rate that was nearly equal to that of normal, un-treated, ASCs. No significant differences were observed under these conditions (Fig. 1B). These data suggest that TMADM-03 could be used to label cells for one hour at a 100 µg-Fe/mL concentration.

### Observation of ATDM and TMADM-03 internalization inside ASCs

**Figure 2:**
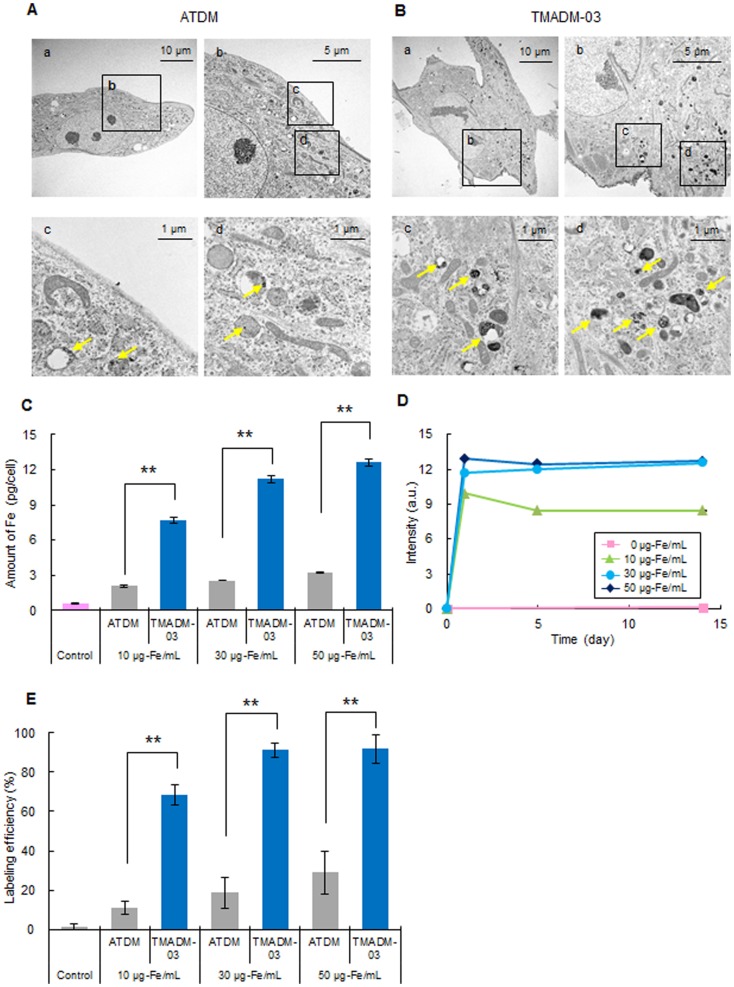
Confirmation of the uptake of ATDM and TMADM-03 by ASCs. A: The images obtained by transmission electron microscopy of ASCs labeled with ATDM (30 µg-Fe/mL) for 1 h at 37°C (a–d). A picture of the cells labeled with ATDM is shown (a). The surface of the cells labeled with ATDM is shown (b). The aggregates of the ATDM internalized by ASCs are shown by yellow arrows in (c) and (d). B: The images obtained by transmission electron microscopy of ASCs labeled with TMADM-03 (30 µg-Fe/mL) for 1 h at 37°C (a–d). A picture of the cells labeled with TMADM-03 is shown (a). The surface of the cells labeled with TMADM-03 is shown (b). The aggregates of the TMADM-03 internalized into ASCs are shown by yellow arrows in (c) and (d). The amount of TMADM-03 internalized in the cytoplasm of ASCs was found to be much higher than that of ATDM. C: The results of the quantitative determinations of the ATDM and TMADM-03 (10, 30, and 50 µg-Fe/mL) internalized into ASCs after a 1 h transduction by measuring the concentration of Fe derived from ATDM or TMADM-03 using ICP-AES. The control (Cont.) shows the amount of Fe normally in ASCs, without labeling by nanoparticles. Significant differences between ATDM and TMADM-03 were confirmed after the transduction at all concentrations. These data are shown as the means ± standard deviation of triplicate values. **P<0.01. D: The changes in the amount of TMADM-03 (0, 10, 30 and 50 µg-Fe/mL) internalized by ASCs after a 1 h transduction for two weeks. The data are shown as the means ± standard deviation of triplicate values. E: The labeling efficiency of TMADM-03 (0, 10, 30 and 50 µg-Fe/mL) for ASCs after a 1 h transduction using MACS Technology.

To detect the internalization of ATDM and the TMADM-03 internalization by ASCs, the cells were transduced with 30 µg-Fe/mL of ATDM or TMADM-03 by a one hour incubation. The SPIO nanoparticles could be observed in ASCs transduced with both ATDM and TMADM-03 using TEM, and these nanoparticles were found in the cell cytoplasm and lysosomes. However, the degree of TMADM-03 incorporation was remarkably higher than that of ATDM (Figs. 2A, B). In addition, as shown in Fig. 2B-b, the surface of ASCs was found to be covered with TMADM-03. These data suggest that both ATDM and TMADM-03 could be transduced into ASCs within one hour of incubation, but the efficiency was markedly higher for TMADM-03.

### Comparison of the uptake of ATDM and TMADM-03 by ASCs

To measure the uptake of ATDM and TMADM-03 by ASCs, the amount of Fe derived from ATDM and TMADM-03 in ASCs was measured using ICP-AES. The amount of Fe was increased in a concentration-dependent manner for both types of nanoparticles. However, the amount of Fe in cells incubated with TMADM-03 was significantly higher (about four-fold) in comparison to that of ATDM at all concentrations (Fig. 2C). The amount of TMADM-03 was confirmed to remain approximately equal for at least 14 days in 30 and 50 µg-Fe/mL labeling conditions (Fig. 2D). In addition, the labeling efficiency was measured by MACS technology, and the efficiency of TMADM-03 was much higher than that of ATDM at all concentrations. Especially, the labeling efficiency of TMADM-03 in 30 and 50 µg-Fe/mL showed more than 90% (Fig. 2E). These data suggest that ASCs could be efficiently labeled with TMADM-03 at the concentration of 30 µg-Fe/mL and maintained the labeling state for at least 14 days.

### Mechanism of TMADM-03 uptake by ASCs

**Figure 3:**
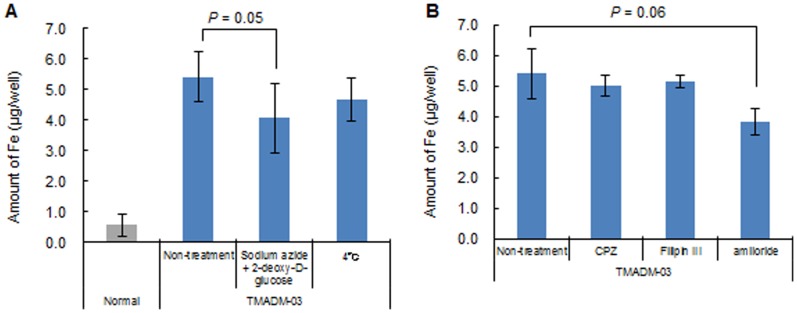
The mechanism of uptake of TMADM-03 by ASCs. A: The effect of endocytosis inhibitors on TMADM-03 internalization in ASCs. Cells were treated with sodium azide and 2-deoxy-D-glucose, or incubated at 4°C. B: The effect of pinocytosis inhibitors on TMADM-03 internalization in ASCs. Cells were treated with chlorpromazine (CPZ), Filipin III, or amiloride. The data are shown as the means ± standard deviation of triplicate values.

To verify the mechanism of uptake of TMADM-03 in ASCs, the cells were treated with endocytosis inhibitors such as sodium azide and 2-deoxy-D-glucose (endocytosis inhibitors), amiloride (an inhibitor of the Na^+^/H^+^ exchanger required for macropinocytosis), filipin III (an inhibitor of caveolae formation) or chlorpromazine (CPZ: an inhibitor of AP-2-mediated clathrin-coated pit formation) at 37°C for one hour (15 min for amiloride). In addition, treatment by incubation at 4°C for one hour was also employed to inhibit endocytosis. The transduction of TMADM-03 was inhibited by the incubation at 4°C and the treatments with sodium azide and 2-deoxy-D-glucose, or amiloride (Figs. 3A, B). These data suggest that the uptake of TMADM-03 into ASCs was mainly dependent on the endocytosis, particularly macropinocytosis.

### Differentiation of ASCs labeled with TMADM-03

**Figure 4:**
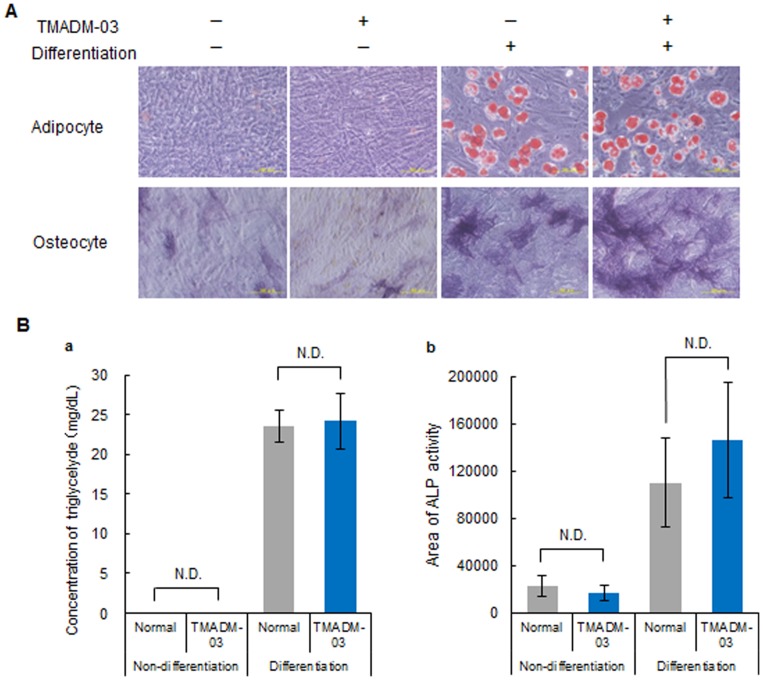
The differentiation capacity of ASCs labeled with TMADM-03. A: The ability of unlabeled ASCs or those labeled with TMADM-03 (30 µg-Fe/mL) to differentiate into adipocytes and osteocytes. The extent of adipogenic differentiation was assessed by Oil Red O staining. Red spherical bodies in the upper figures show lipid droplets produced by the differentiated ASCs (upper). The extent of osteogenic differentiation was assessed by ALP staining. Purple sites in the lower figures show the ALP produced by the differentiated ASCs (lower). B: The degree of differentiation into adipocytes by the concentration (mg/mL) of triglyceride present in the cells (a). The degree of differentiation into osteocytes by the ALP staining area (b). The data are shown as the means ± standard deviation of triplicate values.

To exam the influence of TMADM-03 on the differentiation capacityof ASCs, normal (non-labeled) and labeled ASCs were differentiated into adipocytes or osteoblasts, and then the degree of differentiation in the non-labeled and labeled ASCs was quantitatively compared. The differentiation of ASCs after treatment with TMADM-03 into either adipocytes or osteocytes was observed. There were also no significant differences between the non-labeled and labeled ASCs in terms of the concentration of triglycerides, indicating the degree of adipogenic differentiation (Figs. 4A, B–a). Moreover, similar expression of ALP indicating the degree of osteogenic differentiation was confirmed in the cells incubated with and without the TMADM-03 (Figs. 4A, B–b). These data suggest that TMADM-03 does not affect the differentiation of ASCs.

### Cytokine production from ASCs labeled with TMADM-03

**Figure 5:**
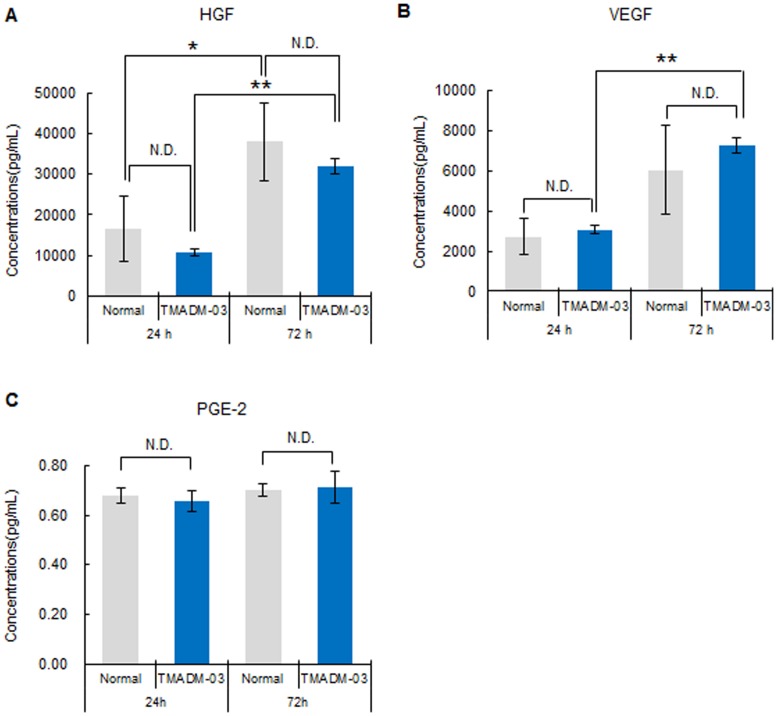
The levels of cytokines produced by ASCs labeled with TMADM-03. A: The concentration of HGF produced by non-labeled (normal) ASCs (1×10^5^) or ASCs labeled with TMADM-03 incubated for 24 and 72 h in the culture medium. B: The concentration of VEGF produced by non-labeled (normal) ASCs (1×10^5^) or ASCs labeled with TMADM-03 incubated for 24 and 72 h in the culture medium. C: The concentration of PGE2 produced by non-labeled (normal) ASCs (1×10^5^) or ASCs labeled with TMADM-03 incubated for 24 and 72 h in the culture medium. The data are shown as the means ± standard deviation of triplicate. *P<0.05, **P<0.01.

To confirm the production of HGF, VEGF and PGE2 from non-labeled ASCs or ASCs labeled with TMADM-03, the levels of these cytokines in the culture medium from ASCs cultured for 24 or 72 h were measured using specific ELISA kits. The production of these cytokines could be confirmed in both non-labeled and labeled ASCs, and no significant differences were observed in the production of any of these cytokines (Fig. 5). These data raised the possibility that the ability of ASCs to produce cytokines could be maintained after labeling with TMADM-03.

### *In vitro* MRI of ASCs labeled with TMADM-03

**Figure 6:**
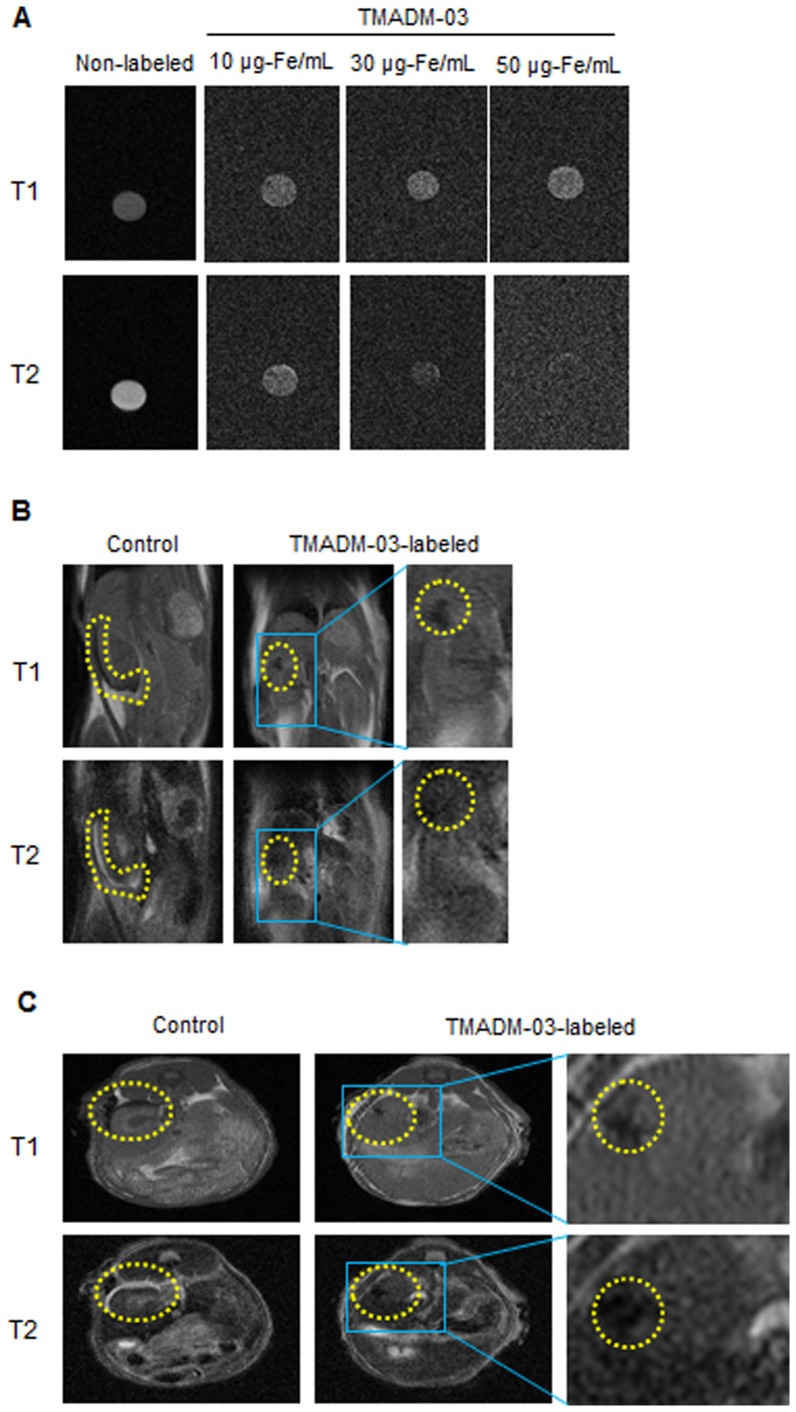
MR imaging of ASCs labeled with TMADM-03 in the kidney capsule. A: *In vitro* MR imaging of unlabeled and labeled ASCs (1×10^6^). T1- (upper) and T2- (lower) weighted images were obtained for unlabeled ASCs and for ASCs labeled with TMADM-03 (10, 30 and 50 µg-Fe/mL). B: *In vivo* MR imaging of unlabelled ASCs (1×10^6^) or the same number of ASCs labeled with TMADM-03 (30 µg-Fe/mL) in a cross-section figure from the back of the mouse. T1- (upper) and T2- (lower) weighted images were obtained for unlabeled ASCs, and for ASCs labeled with TMADM-03 3 hours after transduction. Yellow dotted circles show the transplanted ASCs. C: *In vivo* MR imaging of unlabeled ASCs (1×10^6^) or the same number of ASCs labeled with TMADM-03 (30 µg-Fe/mL) in a cross-section figure from the head of the mouse. T1- (upper) and T2- (lower) weighted images were obtained for unlabeled ASCs and for ASCs labeled with TMADM-03. The yellow dotted circles show the transplanted ASCs. These images were obtained using a 1T MRI instrument (MR Technology).

To examine whether the cells labeled with 10, 30 and 50 µg-Fe/mL of TMADM-03 could be detected by MR imaging, the labeled cells (1×10^6^) were collected in PBS and spun down, then the cell pellet was prepared for the MR analysis in microtubes. The labeled cell pellet could be detected at a lower intensity on both T1 and T2-weighted images in comparison to the unlabeled cell pellet (Fig. 6A). These results suggested that the cells labeled with more than 30 µg-Fe/mL of TMADM-03 could be detected with sufficient contrast for cell visualization by MR imaging.

### MR imaging of ASCs labeled with TMADM-03 in mice

To assess whether images of transplanted ASCs labeled with TMADM-03 could be obtained in mice, the ASCs (1×10^6^) labeled with 30 µg-Fe/mL of TMADM-03 after a one hour incubation were transplanted into the left kidney capsule of a mouse. The MR imaging data of both cross-section figures from the back and head three hour after transplantation showed remarkable decreases in signal intensity on T1 and T2-weighted images at the implanted site in the left kidney of a mouse that was transplanted with ASCs labeled with TMADM-03. On the other hand, in a mouse transplanted with unlabeled ASCs, no decrease in the signal intensity on T2 was observed in the MR imaging results (Figs. 6B C).

**Figure 7:**
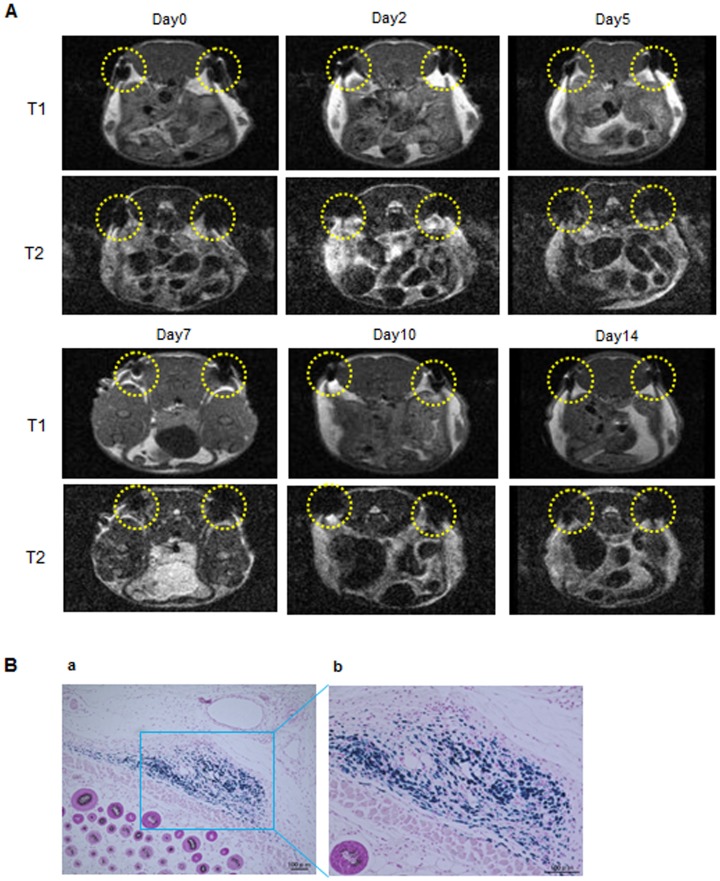
MR imaging of ASCs labeled with TMADM-03 under the skin and Prussian blue staining. A: *In vivo* MR imaging of ASCs (2×10^6^) labeled with TMADM-03 (30 µg-Fe/mL) under the skin in a cross-section figure from the head of the mouse for 14 days. The two yellow dotted circles show the transplanted ASCs labeled with 30 µg-Fe/mL of TMADM-03. These images were obtained using a 1T MRI instrument (MR Technology). B: Prussian blue staining of the transplanted ASCs labeled with TMADM-03.

In addition, to investigate whether the labeled ASCs could be detected for 14 days, the ASCs (2×10^6^) labeled with 30 µg-Fe/mL of TMADM-03 were transplanted under the skin of the back of a mouse at two sites (yellow dotted circles). The ASCs labeled with 30 µg-Fe/mL of TMADM-03 could be traced for at least 14 days after transplantation (Fig. 7A). In addition, to reveal whether the labeled ASCs were alive and did not affect the surrounding tissues or cells, the transplantation sites were treated with PB staining. The blue staining showing the existence of TMADM-03 was confirmed in transplantation sites, and there were no obvious abnormalities such as inflammation in the surrounding tissues or cells (Fig. 7B). These data suggest that the positively charged TMADM-03 was useful as a MR imaging contrast agent for assessing the disposition of transplanted ASCs.

## Discussion

Various SPIO nanoparticles have been developed as contrast agents for MR imaging such as ferucarbotran (Resovist), ferristene (Abdoscan), ferumoxsil (GastroMARK, Lumirenr), ferumoxide (Feridex, Endorem), ferumoxtran-10 (Combidex, Sinerem) and feruglose (Crasican) etc. [11]. Numerous studies have recently revealed these agents to be useful for stem cell labeling for *in vivo* MR imaging [32]–[37]. Among these agents, there have been many studies on ferucarbotran (Resovist, Clivist), which is well known liver contrast agent currently used in the clinical setting [10], [38], [39]. Crabbe et al. revealed that Resovist was useful for labeling mouse mesenchymal stem cells, and that it was superior to Endoderm and Sinerem [4]. However, almost all of these agents have a negative charge coating site on the surface of the SPIO [40]. The surfaces of various cells, including stem cells, normally have many sugar chains whose termini are sialic acid, and these cells therefore have a negative charge on their surface, thus preventing the effective transduction of these cells with the SPIO [41]. We have previously succeeded in performing efficient quantum dots (QDs) labeling for ASCs through an endocytosis pathway using octa-arginine peptides with a positive charge known as cell penetrating peptides (CPPs) [42]. Various CPPs have been identified including the third helix of the homeodomain of antennapedia [43], [44], VP22 herpesvirus protein [45] and the HIV-Tat protein [46]. Most of these peptides have a positive charge derived from amino acids such as arginine and lysine. In this study, we demonstrated that TMADM-03, which has a positive charge, can label ASCs more efficiently than ATDM which is a major component of ferucarbotran that has a negative charge.

We have already reported that the zeta voltages of ATDM and TMADM-03 were -15 mV and +2.0 mV, respectively [23]. When ASCs were transduced with TMADM-03 during a one hour incubation, the surface of ASCs was observed to be covered with TMADM-03 by a TEM analysis. The successful uptake of TMADM-03 by the ASCs was therefore thought to have occurred. The same phenomenon was not found in the case of ATDM. ATDM was thought to incidentally come into contacted with the surface of ASCs, and to subsequently be incorporated into the ASCs. As a result, about a four-fold higher uptake of TMADM-03 in comparison to ATDM was observed by ICP-AES in the present day. According to previous reports, positively charged substrates, such as protamine, can effectively increase the efficiency of transduction of magnetic nanoparticles into cells. Huang et al. reported that an approximately two-fold higher uptake of ATDM was observed when it was complexed with protamine in comparison to the uptake of ATDM alone [39]. In addition, Balakumaran reported that labeling by ferumoxide complexed with protamine did not affect the stemness of bone marrow mesenchymal stem cells [34]. However, the efficiency of uptake of ATDM complexed with protamine is assumed to be lower than that of TMADM-03, and the influence of the released protamine on other types of stem cells remains unclear.

Slight cytotoxicity was observed in ASCs transduced with TMADM-03 during a 24 h incubation. However, no cytotoxicity was observed after one hour of incubation at a concentration of up to 100 µg/mL of TMADM-03, and the ASCs labeled with TMADM-03 under these non-cytotoxic conditions exhibited growth equivalent to that of normal ASCs, and could be successfully detected by MRI. The capacity of these cells to differentiate in adipocytes and osteocytes was not affected, and the ability of labeled ASCs to produce cytokines such as HGF, VEGF and PGE2, which are thought to be important for regenerative effects, was maintained after the labeling with TMADM-03. Moreover, the transduction of TMADM-03 into ASCs was inhibited by sodium azide and 2-deoxy-D-glucose (endocytosis inhibitors), and amiloride (a macropinocytosis inhibitor). Although the uptake mechanism of TMADM-03 had previously been unknown, our data indicate that the uptake pathway of TMADM-03 is thought to be mainly dependent on the endocytosis, partially macropinocytosis. These data suggest that TMADM-03 can be a safe and efficient MR contrast agent that can be used to label stem cells for clinical applications.

Using a 1T MR imaging system for small animals, we demonstrated that the ASCs labeled with TMADM-03 could be detected both *in vitro* and *in vivo*. As shown in Fig. 6A, the MR images of the pellet of ASCs labeled with TMADM-03 in a microtube had low signal, and a negative contrast effect could be confirmed. When ASCs labeled with TMADM-03 were transplanted under the skin and the left kidney capsule of a mouse, a negative effect on T2-weighted contrast images could be detected when TMADM-03 was used (Figs. 6B, 6C, 7A). Furthermore, the inflammatory state such as induced by the cell death could not be observed in the surrounding area of the transplantation of ASCs labeled with TMADM-03. These data suggest that TMADM-03 can be used as a contrast agent both *in vitro* and *in vivo* for the MR imaging of stem cells, and raise the possibility that TMADM-03 can provide insights into the location and accumulation of transplanted stem cells in tissues or organs deep in the body.

In conclusion, we investigated whether TMADM-03, which was previously developed by our group, could be used to label ASCs as a MR imaging contrast agent. No cytotoxicity was observed in the ASCs transduced with a concentration of up to 100 µg-Fe/mL of TMADM-03 for a one hour transduction time. The transduction efficiency of TMADM-03 into ASCs was about four-fold higher than that of ATDM, which is a major component of ferucarbotran (Resovist), a clinically-used contrast agent. Of note, the labeling level was maintained for at least two weeks. ASCs labeled with TMADM-03 were confirmed to be able to differentiate into both adipocytes and osteocytes to the same extent as non-labeled ASCs. In addition, the ability of ASCs labeled with TMADM-03 to product cytokines (HGF, VEGF and PGE2) was maintained. The ASCs labeled with TMADM-03 could be imaged with good contrast using a 1T MR imaging system when the labeled ASCs were transplanted into the left kidney capsule of a mouse. Together, these data suggest that TMADM-03 can be utilized as a MR imaging contrast agent for tracking transplanted stem cells.
